# Supplementary figures and images for: Individual Differences in Dynamic Functional Brain Connectivity across the Human Lifespan
Source: PLoS Comput Biol. 2016 Nov 23;12(11):e1005178. doi: 10.1371/journal.pcbi.1005178 (PMC5120784; doi:10.1371/journal.pcbi.1005178)

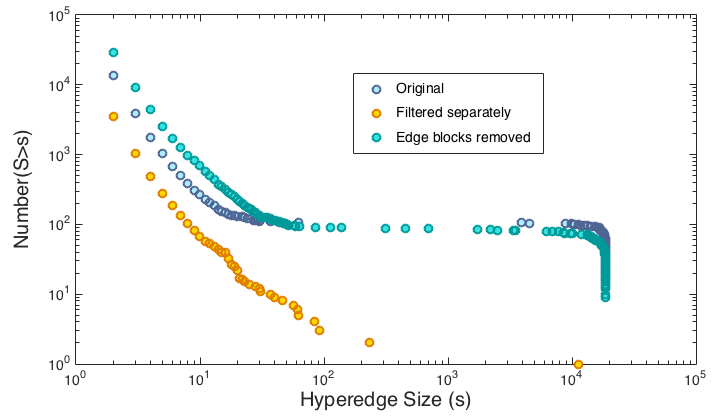

Supplement: S1 Fig — Cumulative size distributions for the original age-memory data set (with no changes to remove effects of the edges) and two methods for removing potential effects from the edges. The “edge blocks removed” method is used in all analyses in the main text. (TIF) [file pcbi.1005178.s001.tif]

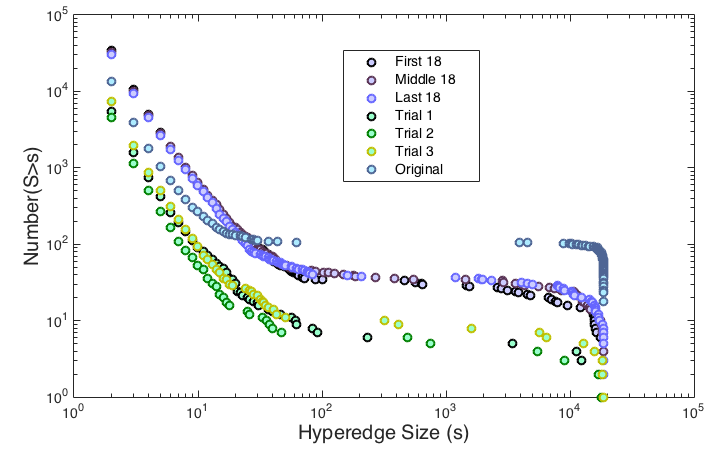

Supplement: S2 Fig — Cumulative size distributions for two different methods for separating edge effects. In the trial-by-trial method, hypergraphs are constructed separately for each trial, while in the 18-split analysis, hypergraphs are constructed from the first, middle, or last 18 edge time series data points. (TIF) [file pcbi.1005178.s002.tif]

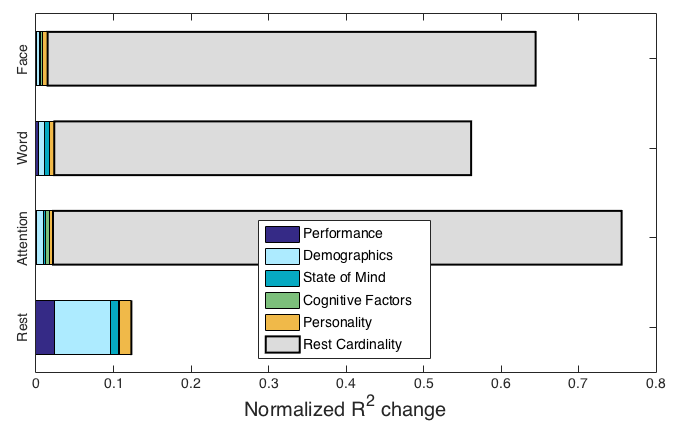

Supplement: S3 Fig — Normalized R2 changes with respect to task-specific hypergraph cardinality for each of the four task-specific hypergraphs. Rest-specific hypergraph cardinality is included as an independent variable for the other three tasks and is the only significant predictor, which is denoted with a bold outline. (TIF) [file pcbi.1005178.s003.tif]
