# Supplementary material for: Individual Differences in Dynamic Functional Brain Connectivity across the Human Lifespan
Source: PLoS Comput Biol. 2016 Nov 23;12(11):e1005178. doi: 10.1371/journal.pcbi.1005178 (PMC5120784; doi:10.1371/journal.pcbi.1005178)
Supplement: S1 Table — Categories containing measures of interest (42). For the state of mind measures, (Y/N) indicates measures where participants were asked whether they had performed the activity in the past 24 hours. (PDF) [file pcbi.1005178.s004.pdf]

Performance (Word)	Demographics	Personality	Cognitive Factors	State of Mind
Criterion shift score	Age	PANAS (6)	OSIQ-S/O	Arrival time
Liberal Dprime	Sex	Big 5 (5)	VVQ-W/P	Meal (hours since)
Conservative Dprime	Education (years)	BIS/BAS (4)	Need for cognition	Hours of sleep
Overall Dprime	Dominant hand		SBCSQ visual	Physical/mental comfort
			SBCSQ verbal	Beck Depression Inventory
			Paper folding	Alcohol (Y/N)
			Card rotation	Exercise (Y/N)
				Smoking (Y/N)
				Caffeine (Y/N)
