# Supplementary material for: Individual Differences in Dynamic Functional Brain Connectivity across the Human Lifespan
Source: PLoS Comput Biol. 2016 Nov 23;12(11):e1005178. doi: 10.1371/journal.pcbi.1005178 (PMC5120784; doi:10.1371/journal.pcbi.1005178)
Supplement: S2 Table — Categories containing measures of interest. For the state of mind measures, (Y/N) indicates measures where participants were asked whether they had performed the activity in the past 24 hours. (PDF) [file pcbi.1005178.s005.pdf]

Performance	Demographics	Personality	Cognitive Factors	State of Mind
Attention CS<br>Face memory CS<br>Attention Dprime<br>Face memory Dprime	Military rank	EPQ-R (4)	Working memory<br>Vocabulary test	MSW/MSF<br>PTSD Score<br>PTSD (Y/N)<br>Concussion score<br>Concussion 5 inventory
