# Supplementary material for: Individual Differences in Dynamic Functional Brain Connectivity across the Human Lifespan
Source: PLoS Comput Biol. 2016 Nov 23;12(11):e1005178. doi: 10.1371/journal.pcbi.1005178 (PMC5120784; doi:10.1371/journal.pcbi.1005178)
Supplement: S3 Table — Categories containing measures of interest. For the state of mind activity measures, yes indicates measures where participants were asked whether they had performed the activity in the past 24 hours. Questions about daily, weekly, and monthly amounts of activity, including whether activity in the past 24 hours were more or less than usual were also recorded for all (Y/N) state of mind activities in the age-memory study. (PDF) [file pcbi.1005178.s006.pdf]

Performance	Demographics	Personality	Cognitive Factors	State of Mind
Hit rates	Height	Distracted		Stressed (Y/N)
Failure rates	Weight	Motivated		Days since period
Reaction time	Contraceptive use			Usual hours of sleep
	Children (Y/N)			Drugs past 48h (Y/N)
	Number of children			MMSE (dementia)
